# A novel method for the storage and transport of biological samples of therapeutic proteins prior to the detection of analytes using ELISA

**DOI:** 10.1038/s41598-021-88180-0

**Published:** 2021-04-22

**Authors:** Lei Wang, Lixiong Liu, Xiaoping Hong, Dongzhou Liu, Zeneng Cheng

**Affiliations:** 1 Department of Rheumatology and Immunology, The Second Clinical Medical College, Jinan University (Shenzhen People’s Hospital), Shenzhen, 518020 China; 2 Integrated Chinese and Western Medicine Postdoctoral Research Station, Jinan University, Guangzhou, 510632 China; 3 Research Institute of Drug Metabolism and Pharmacokinetics, School of Xiangya Pharmaceutical Sciences, Central South University, Changsha, 410013 Hunan China

**Keywords:** Pharmacokinetics, Clinical pharmacology

## Abstract

Therapeutic proteins have exhibited promising clinical applications in the diagnosis and treatment of some diseases. Prior to the detection of analytes using enzyme-linked immunosorbent assay, biological samples of therapeutic proteins are conventionally frozen at temperatures ranging from − 20 to − 80 °C to increase the stability of analytes. However, therapeutic proteins destabilization and aggregation may occur during the frozen storage or the freeze-thawing step. In this work, an effective method was proposed to freeze-dry therapeutic protein samples to allow subsequent storage or transport of samples without freezing them. This new method was validated with quality control samples of adalimumab and etanercept, and it was also used in the bioanalysis of adalimumab and etanercept in pharmacokinetic (PK) studies. Adalimumab and etanercept were stable for 14 days at 4 °C after being prepared and stored using the new method, with detection that was accurate and repeatable. Studies of adalimumab and etanercept in animals and humans showed that the PK parameters of the analytes stored with the new method were consistent with those of analytes stored using the conventional method. This effective method will be attractive for facilitating the storage and transport of plasma samples containing therapeutic proteins.

## Introduction

Therapeutic proteins are widely used in the diagnosis and treatment of cancers and immunological-based disorders^1,2^. To date, almost 60 types of new therapeutic proteins have been tested in clinical trials^3^. To investigate the pharmacokinetic (PK) properties of new drugs in preclinical or clinical studies, biological samples including plasma and serum are collected to measure the concentrations of drugs and subsequently profile the disposition of drugs in vivo^4–6^.

Therapeutic proteins are often frozen at low temperatures ranging from − 20 to – 80°C^7^. The advantages of frozen storage include increased sample stability and decreased microbial growth^8,9^. However, under these storage conditions, protein destabilization and aggregation may occur during storage or during the freeze-thawing step^10,11^. Different storage methods are desired that can improve the accuracy of analyte detection and reduce drug decomposition.

The use of dried blood spots and dried plasma spots has recently been proposed for the storage of blood and plasma samples^12,13^. Samples stored by these methods can be maintained at ambient temperature and conveniently transported. Nonetheless, both technologies are imperfect, and samples can be influenced by the temperature and humidity of the environment that was present during spotting, the volume of blood or plasma that was used, and other factors^14^.

To simplify the process of sample storage and transport, we developed a novel freeze-drying device as well as a new sample storage method. The device and method have been validated by applying them to the storage of two quality control (QC) samples including adalimumab and etanercept. Subsequently, the proposed method was successfully applied to PK studies of adalimumab and etanercept in animals and humans.

## Methods

### Materials

Adalimumab (Humira, 40 mg/0.8 ml) was purchased from the manufacturer (Vetter Pharma-Fertigung GmbH & Co. KG, Germany), and etanercept (Enbrel, 25 mg) was purchased and used as received from the provider (Pfizer, New York, NY, USA). Recombinant human TNF-α (Peprotech) was used to capture the adalimumab or etanercept by being immobilized on the solid phase surface of ninety-six-well plates (Greiner, Germany). A 5% solution of nonfat dried milk (Dingguo Changsheng Biotechnology, China) was dissolved in phosphate-buffered saline (PBS) (Dingguo Changsheng Biotechnology) for sealing the solid phase surface of each well. 0.5% Tween-20 (Damao Chemical Reagent Factory, China) was added into PBS to prepare a wash solution. Horseradish peroxidase (HRP)-goat anti-human IgG (H + L) conjugate (ABclonal Technology, Woburn, MA, USA) was used for detecting adalimumab or etanercept. Tetramethyl benzidine (TMB) (Solarbio, China) was used as the substrate solution, and 2 mol/L hydrogen chloride (Sinopharm Chemical Reagent, China) were prepared in the laboratory to be used as a stop solution.

### Novel method for sample storage and preparations

#### Freeze-drying device

**Figure 1:**
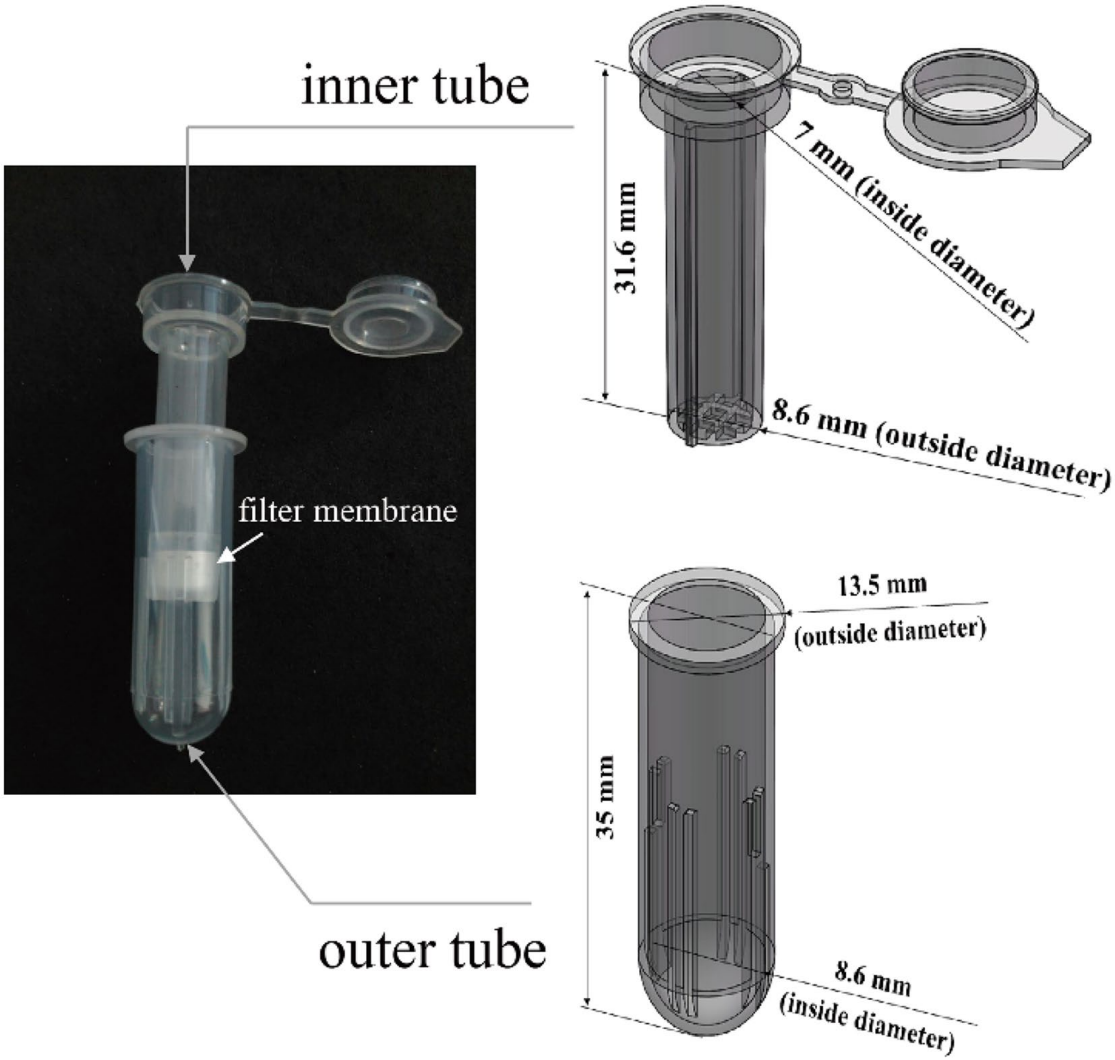
The structure the freeze-drying device. The proposed device consisted of two tubes. The inner tube was used for intercepting the analytes with a membrane, and the outer tube was used for reserving the filtered samples. The inner tube was freeze-dried in a vacuum freeze dryer. The volume of the sample stored in each tube was 200 µL.

The novel freeze-drying device consisted of two tubes. The inner tube was used for intercepting the analytes with a membrane, and the outer tube was used for reserving the filtered samples (Fig. 1). The heights of the tubes were 31.6 mm and 35 mm, respectively. The diameters of the inner tube and outer tube were 7 mm and 8.6 mm, respectively. The membrane placed at the bottom of the inner tube evenly dispersed the samples and efficiently filter them. After adding 50 µL 5% nonfat dried milk, 200 µL plasma or serum samples were placed into the inner tube and ultrasonically blended for 5 min. The filtered sample then flowed out from the inner tube through the "#" -shaped bottom pore and was reserved in the outer tube. Eventually, the inner tube was freeze-dried in a vacuum freeze dryer. The volume of the sample stored in each tube was 200 µL.

#### Freeze-drying process

The samples were previously frozen at – 40 °C in the freeze dryer for 3 h. Then the vacuum pump began to work, and all samples were heated to – 37 °C. The freeze drier was programmed to slowly increase the temperature until it reached 20 °C. After completing all programmed settings, the freeze-dried tubes were removed and stored in low-gas-permeable sealed bags at 4℃ until analysis.

#### Sample preparations

To each freeze-dried tube, 800 μL of normal saline was added, followed by soaking for 30 min, and then the contents of the tube were sonicated for 10 min. After all tubes were centrifuged at 15,700 × *g* for 10 min at 4 °C, the supernatants were analyzed by enzyme-linked immunosorbent assay (ELISA) to detect the concentrations of adalimumab and etanercept.

### Conventional method for sample storage and preparations

As the biological samples (plasma or serum) of adalimumab or etanercept were collected, they were transferred to polyethylene tubes and then stored in a low-temperature freezer at – 80 °C until the time of the assay. Prior to the detection of adalimumab or etanercept, 200 μL of thawed sample was transferred to another tube and then centrifuged at 440 × *g* for 5 min. Then, 20 μL of the supernatant was analyzed by the ELISA method.

### Analytical methods

#### ELISA detection of adalimumab

The 96-well plates were coated with 1 μg/mL recombinant human TNF-α (100 µl/well) on their solid phase surface and were then stored at 4 °C for 12 h. After PBST was used to wash the wells 3 times, the wells were coated with blocking reagent (5% nonfat dried milk in PBST, 250 μL/well) at 37 °C for 2 h. The serum samples, which were diluted in 1% nonfat dried milk solution, were added to the 96-well plate (50 μL/well) following removal of the blocking buffer and washing the wells with PBST for three times, and then the plate was placed in an incubator at 37 °C for 1 h. Afterward, PBST was used to wash the wells 5 times, and 1 μg/mL HRP-goat anti-human IgG (H + L) conjugate was added (50 μL/well). The plates were subsequently incubated at 37 °C for 30 min for detection of adalimumab. Next, after 5 washes, TMB substrate was added (100 μL/well) and allowed to develop for 10 min. Then, the stop solution (1 M sulfonic acid) was added to stop the reaction (50 μL/well). The OD values were read at 450 nm within 15 min after adding stop solution. All measurements were performed in duplicate. A standard concentration curve ranging from 31.25 to 1000 pg/mL was prepared using an adalimumab standard.

#### ELISA detection for etanercept

The detection of etanercept was performed with ELISA method^15^. First, 1.5 μg/mL recombinant human TNF-α was coated on the solid phase surface of the 96-well plates (100 μL/well), and they were stored overnight at 4 °C. Second, after coating with the TNF-α, the wells were washed three times with PBST, and blocked with 5% nonfat dried milk/PBST (250 μL/well) in a stationary incubator at 37 °C for 2 h. Third, after removal of the blocking solution and washing the wells three times with PBST, plasma samples (diluted in 1% nonfat dried milk/PBST) were added to the plate (50 μL/well), which was then incubated at 37 °C for 1 h. Fourth, the wells were washed five times with PBST, and etanercept was detected with 1 μg/mL HRP-goat anti-human IgG (H + L) conjugate (50 μL/well) by incubating at 37 °C for 30 min. Finally, after washing the wells five times with PBST, color development was performed by the addition of 100 μL TMB into each well, and then stopping the reaction with 1 mol/L sulfonic acid (50 μL/well). Optical density was measured at 450 nm with a correction at 630 nm. This method detected the free etanercept, and all measurements were performed twice. A standard curve was prepared with etanercept ranging from 6.25 to 500 pg/mL.

### Validations of the new storage method

#### Validation with QC samples of adalimumab

QC samples of adalimumab were stored using either the new method or the conventional method. The ELISA method was used to detect adalimumab in all samples. The accuracy, precision, and stability of samples prepared with the two storage methods were evaluated using a modified method^16^.

Standard working solutions of adalimumab were prepared by diluting stock solutions with nonfat dry milk to six different concentration levels including 312.5, 625, 1250, 2500, 5000, and 10,000 pg/mL. Standard working solutions were added to plasma from rats or healthy human volunteers to achieve the following calibration standard concentrations: 31.25, 62.5, 125, 250, 500, and 1000 pg/mL. The QC samples were similarly prepared at three concentration levels of 90, 600, and 750 pg/mL (n = 5, at each concentration). All calibration standard samples and QC samples were freshly prepared daily.

Intra- and inter-day accuracy and precision were evaluated by measurement of QC samples at three concentration levels (90, 600, and 750 pg/mL) with five replicates. The concentration of each sample was calculated according to the calibration curve prepared the same day. Accuracy was defined as the relative deviation in the determined concentration of a standard from that of its nominal concentration, and the precision of the analyte is expressed as the relative standard deviation (RSD). The stability of adalimumab was evaluated by measurement of three replicate of QC samples after they were separately stored using either the new or the conventional method for 7 d and 14 d.

#### Validation with QC samples of etanercept

QC samples of etanercept were stored using either the new method or the conventional method. The accuracy, precision, and stability of the differently stored samples were evaluated by ELISA. The standard calibration concentrations were 6.25, 12.5, 25, 50, 100, 250, and 500 µg/mL, and the QC samples were prepared at three concentration levels of 12.5, 100, and 400 µg/mL. The intra- and inter-day accuracy, precision, and stability of etanercept were calculated in the same manner as those used in the validation with the QC samples of adalimumab.

### PK studies

#### Ethics

Animal studies were approved by the Animal Ethics Committee of the School of Pharmaceutical Sciences of Central South University (Changsha, China). All experiments were conducted in accordance with the ARRIVE guidelines as well as the National Institutes of Health Guide for the Care and Use of Laboratory Animals.

The trials of adalimumab in patients and the trials of etanercept in healthy volunteers and patients were both approved by the Ethics Committee of the School of Pharmaceutical Sciences of Central South University. Written informed consent was obtained from all individual participants included in the study in accordance with the Good Clinical Practice guidelines published by the National Medical Products Administration of China.

#### PK study of adalimumab in rats

For the PK study, 0.5 ml blood was collected from each of 10 rats that subsequently received one subcutaneous injection of 0.5 mg adalimumab. After the single-dose administration of adalimumab, 0.5 ml blood was collected before and at 0.5, 1, 2, 6, 12, 24, 48, 96, 168, 264, 336, and 504 h. After the blood was collected, the serum samples were centrifuged at 780 × *g* for 10 min and maintained at 4℃ for 30 min. All serum samples were divided into two groups to be separately stored using the new or conventional method until analysis.

#### PK study of adalimumab in patients with rheumatoid arthritis

Six Chinese male patients with rheumatoid arthritis (RA), between 27 and 39 years old and the body weights from 60 to 75 kg, were enrolled to receive multiple injections of adalimumab. Inclusion in the study was made according to a diagnosis of RA based on rigid criteria^15^. Exclusion criteria included the following: complete tetanus; serious disease of the heart, liver, kidney, or endocrine system; infection (acute or chronic); HbsAg-positive status; HIV-positive status; and a history of tuberculosis or a positive tuberculosis skin test.

All patients received consecutive subcutaneous injections of 40 mg adalimumab once every two weeks in the abdomen. 2 mL blood samples were collected in heparinized centrifuge tubes before the fifth, sixth, and seventh administration. The plasma samples were separated by centrifugal force at 3500 rpm for 10 min, and then samples were divided into two groups to be separately stored using the new or conventional method until analysis.

#### PK study of etanercept in healthy volunteers

Six Chinese healthy volunteers including 3 males and 3 females, between 21 and 31 years old, and weighting from 50 to 63 kg, were enrolled to receive single subcutaneous injections of etanercept. Exclusion criteria included any clinically significant medical history or physical findings, presence of pregnancy or lactation, blood or blood product donation within 30 days of medication administration, serious disease of the heart, liver, kidney, or endocrine system, prior anti-TNF-α therapy, infection (acute or chronic), HbsAg-positive status, HIV-positive status, a history of tuberculosis or a positive tuberculosis skin test, and psychotic, emotional or intellectual problems likely to limit the validity of the consent^15^.

Prior to the subcutaneous injection of 50 mg etanercept, 5 mL blood was collected from the 6 healthy volunteers. After administration of etanercept, 5 mL blood samples were collected in heparinized centrifuge tubes at 2, 4, 8, 12, 24, 36, 48, 72, 120, 168, 336, 504, 672, 744, and 888 h. All plasma samples were separated by centrifugation at 3500 rpm for 10 min, and then, the samples were stored separately using either the new method or the conventional method until analysis.

#### PK study of etanercept in patients with ankylosing spondylitis

Six Chinese male patients with ankylosing spondylitis (AS), between 23 and 52 years old and weighting from 60 to 84 kg, were enrolled to receive multiple injections of etanercept. Inclusion into the study was made according to a diagnosis of AS based on rigid criteria^15^. Exclusion criteria included the following: complete tetanus; serious disease of the heart, liver, kidney, or endocrine system; infection (acute or chronic); HbsAg-positive status; HIV-positive status; and a history of tuberculosis or a positive tuberculosis skin test.

All patients received eight consecutive subcutaneous injections of 50 mg etanercept once a week in the abdomen. 2 mL blood samples were collected in heparinized centrifuge tubes before the fifth, sixth, and seventh administration. The plasma samples were separated by centrifugal force at 3500 rpm for 10 min, and then samples were separately stored using the new or conventional method until analysis.

## Results

### Validation with QC samples of adalimumab

The new method was validated with QC samples of adalimumab (Table 1). The detection of QC samples was proved to be accurate and repeatable with intra- and inter-day precision (RSD) within 15% and accuracy between 80–120%. The results were consistent with the analysis of the same samples stored with the conventional method. All the above results indicate that adalimumab was stable when QC samples were stored with either the new method or the conventional method.

**Table 1:** Intra- and inter-day accuracy and precision, and stability of adalimumab.

Sample storage methods	Nominal concentration (pg/mL)	Intra-assay		Inter-assay		Short-term stability (7 days)		Long-term stability (14 days)	
		RSD (%)	Accuracy (%)	RSD (%)	Accuracy (%)	RSD (%)	Accuracy (%)	RSD (%)	Accuracy (%)
New method	90	4.0	94.3	7.3	95.3	5.2	92.4	5.4	89.6
	600	5.9	102.1	8.7	105.0	6.1	98.4	5.1	92.5
	750	4.3	100.2	4.6	98.8	3.8	103.8	3.5	94.2
Conventional method	90	8.4	100.8	9.1	103.2	5.8	109.0	7.1	92.1
	600	2.6	94.1	6.9	100.5	3.9	94.3	4.4	98.8
	750	3.3	95.7	5.5	100.3	4.2	97.5	4.9	101.5

In addition, when QC samples were stored with either method for 7 d, the stability of adalimumab was satisfactory for the RSD, and the accuracy of the detection was acceptable. The stability of adalimumab was confirmed when the samples were stored with either method for 14 d.

### Validation with QC samples of etanercept

As shown in Table 2, when etanercept QC samples were stored using either the new or conventional method, the analysis of etanercept was accurate and repeatable, with the RSD within 15% and the accuracy between 80–120%. In addition to good precision and accuracy, good stability of etanercept was also achieved for the RSD and the accuracy of the detection was acceptable when QC samples were stored with either method for 7 d. The stability of etanercept was confirmed when the samples were stored with either method for 14 d.

**Table 2:** Intra- and inter-day accuracy and precision, and stability of etanercept.

Sample storage methods	Nominal concentration (pg/mL)	Intra-assay		Inter-assay		Short-term stability (7 days)		Long-term stability (14 days)	
		RSD (%)	Accuracy (%)	RSD (%)	Accuracy (%)	RSD (%)	Accuracy (%)	RSD (%)	Accuracy (%)
New method	12.5	4.1	96.7	9.5	109.4	7.8	94.6	10.5	93.1
	100	2.8	100.4	7.4	105.9	7.4	106.5	8.4	95.2
	400	2.6	98.9	8.6	98.0	10.6	101.2	6.6	99.5
Conventional method	12.5	11.9	93.4	11.1	94.0	8.4	104.0	10.3	95.6
	100	4.2	90.9	6.2	95.9	7.5	106.3	8.4	101.8
	400	3.0	91.1	8.1	99.7	9.1	99.4	7.8	100.0

### Applications

#### PK study of adalimumab in rats

**Figure 2:**
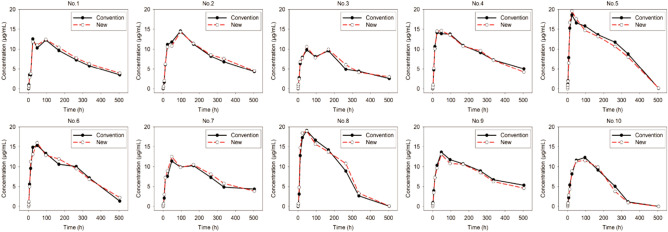
The concentration–time profiles for 10 rats (NO. 1–10) after they receive one subcutaneous injection of 0.5 mg adalimumab. 0.5 ml blood was collected before and at 0.5, 1, 2, 6, 12, 24, 48, 96, 168, 264, 336, and 504 h. Serum samples were separately stored using the new (red symbols and line) or the conventional method (black symbols and line) until analysis. All samples were detected with the ELISA method.

Figure 2 shows the concentration–time profiles for 10 rats after they receive a single subcutaneous injection of 0.5 mg/kg adalimumab. Consistent results were achieved in the concentration detection of samples after they were stored with either the new method or the conventional method. The relative deviation of the detection of both samples was within 15% at each timepoint. The non-compartmental parameters of adalimumab stored with the new method were also consistent with those stored with the conventional method (Table 3). As a macromolecular protein, adalimumab was absorbed slowly in rats, and the T_max_ of adalimumab was approximately 60.00 h. Adalimumab was widely distributed, with an apparent distribution volume reaching 22.26 L in rats. Adalimumab is a humanized monoclonal antibody that is eliminated slowly in vivo, with an elimination rate of 161.00 h. Similar results were also observed in other PK studies of adalimumab in rats.

**Table 3:** The PK parameters of adalimumab for 10 rats after they receive one subcutaneous injection of 0.5 mg adalimumab.

Parameters^a^	Sample storage methods	
	New method	Conventional method
t_1/2_ (h)	161.00 ± 98.38	183.11 ± 123.82
T_max_ (h)	60.00 ± 25.92	50.40 ± 26.41
C_max_ (μg/mL)	14.37 ± 3.01	14.16 ± 2.94
AUC_last_ (μg/mL*h)	4104.96 ± 744.18	4073.92 ± 780.96
AUC_inf_ (μg/mL*h)	4961.62 ± 1059.01	5114.28 ± 1298.62
MRT_inf_ (h)	289.20 ± 106.14	314.63 ± 135.34
Vz/F (L)	22.26 ± 12.38	24.42 ± 15.21
Cl/F (L/h)	0.11 ± 0.03	0.10 ± 0.03

^a^Each parameter was performed with a variance test, and the *P* value was bigger than 0.05.

#### PK study of adalimumab in patients with rheumatoid arthritis

Prior to each patient receiving the fifth, sixth, and seventh administration of 40 mg adalimumab, the concentration of adalimumab in vivo was detected (Table 4). Consistent results were achieved in the concentration detection of samples after they were stored uisng either the new method or conventional method. The relative deviation of the detection of samples was within 15% at each timepoint. There was significant individual difference in the concentration of adalimumab after each administration of adalimumab. Regarding the blood concentration of adalimumab in one patient, there were no significant differences before the fifth, sixth, and seventh administration of adalimumab. This implied that the blood concentration in vivo seemed to reach a steady state after administration of consecutive subcutaneous injections of adalimumab.

**Table 4:** The concentration of adalimumab in patients with RA before the fifth, sixth, and seventh administration of 40 mg adalimumab.

No	Before 5th administration			Before 6th administration			Before 7th administration		
	New method	Conventional method	Accuracy (%)	New method	Conventional method	Accuracy (%)	New method	Conventional method	Accuracy (%)
Ada-1	523.4	512.5	102.1	582.9	606.3	96.1	540.3	527.3	102.5
Ada-2	736.2	765.3	96.2	875.2	859.3	101.9	751.5	773.3	97.2
Ada-3	304.5	294.2	103.5	345.5	324.5	106.5	233.1	240.4	97.0
Ada-4	164.6	186.5	88.3	126.7	134.0	94.6	186.4	207.4	89.9
Ada-5	230.1	236.7	97.2	267.2	251.3	106.3	283.8	262.1	108.3
Ada-6	387.1	374.5	103.4	373.3	385.1	96.9	406.9	392.4	103.7

#### PK study of etanercept in healthy volunteers

**Figure 3:**
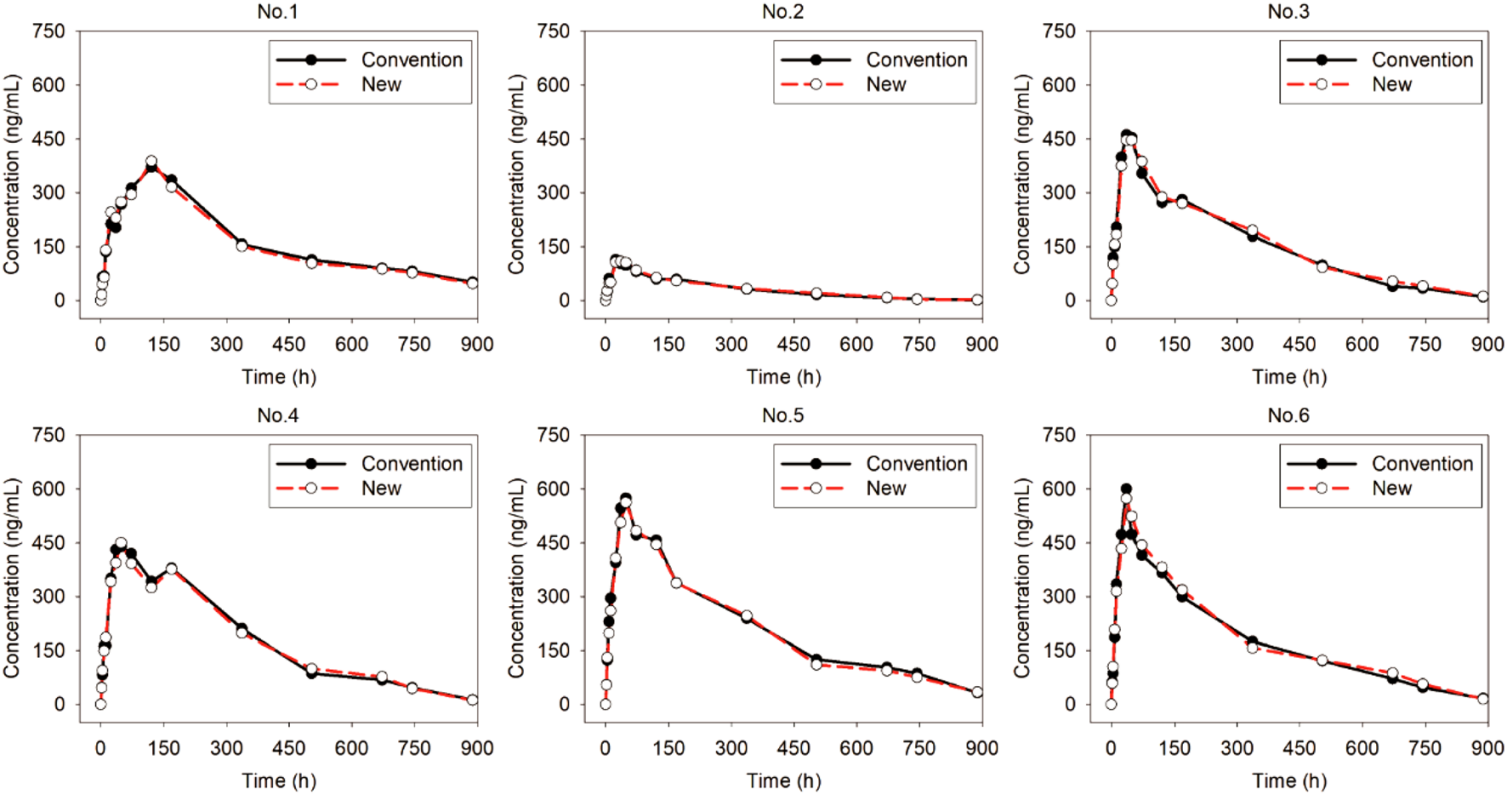
The concentration–time profiles for 6 healthy volunteers (NO. 1–6) after they receive one subcutaneous injection of 50 mg etanercept. 5 mL blood sample was collected in heparinized centrifuge tubes at 2, 4, 8, 12, 24, 36, 48, 72, 120, 168, 336, 504, 672, 744, and 888 h. Serum samples were separately stored using the new (red symbols and line) or the conventional method (black symbols and line) until analysis. All samples were detected with the ELISA method.

Concentration–time profiles of etanercept are illustrated after healthy volunteers receive a single subcutaneous injection of 50 mg etanercept (Fig. 3). Consistent results were achieved in the concentration detection of samples, which were stored with either the new method or conventional method. The relative deviation of the detection of both samples was within 15% at each timepoint. The non-compartmental parameters of etanercept stored with the new method were also consistent with those stored using the conventional method (Table 5). No significant differences were observed for samples prepared using the two methods.

**Table 5:** The PK parameters of etanercept for six healthy volunteers after they receive one subcutaneous injection of 50 mg etanercept.

Parameters^a^	Sample storage methods	
	New method	Conventional method
t_1/2_ (h)	145.32 ± 72.07	174.71 ± 109.33
T_max_ (h)	56.00 ± 31.90	52.00 ± 34.50
C_max_ (μg/mL)	421.93 ± 169.00	427.13 ± 175.35
AUC_last_ (μg/mL*h)	133,534.31 ± 54,189.32	133,830.22 ± 55,361.61
AUC_inf_ (μg/mL*h)	138,932.98 ± 57,074.57	141,268.57 ± 59,629.49
MRT_inf_ (h)	296.59 ± 57.53	307.31 ± 92.10
Vz/F (L)	118.06 ± 137.51	108.66 ± 73.90
Cl/F (L/h)	0.56 ± 0.60	0.57 ± 0.64

^a^Each parameter was performed with a variance test, and the P value was bigger than 0.05.

As a macromolecular protein, the absorption of etanercept is slow, with a T_max_ of 56.00 h. The value of apparent distribution volume of etanercept was about 118.06 L, which implied that etanercept was widely distributed in vivo. As a humanized fusion protein, etanercept would encounter a slow metabolism in an analogous way as most proteins or peptides do in vivo, and thus, the elimination rate of adalimumab is as slow as to 145.32 h. Similar results were also observed in other PK studies of etanercept.

#### PK study of etanercept in patients with ankylosing spondylitis

The concentration of etanercept in patients with ankylosing spondylitis was detected before patients received the fifth, sixth, and seventh administration of 50 mg etanercept (Table 6). Consistent results were achieved in the concentration detection of samples after they were stored with either the new method or conventional method. The relative deviation of the detection of samples was within 15% at each timepoint. There was no significant difference among the in vivo concentrations of etanercept before the fifth, sixth, and seventh administration of etanercept, which implied that the blood concentration of etanercept in vivo reached a steady state after patients received consecutive subcutaneous injections of etanercept.

**Table 6:** The concentration of etanercept in patients with AS before the fifth, sixth, and seventh administration of 50 mg etanercept.

No	Before 5th administration			Before 6th administration			Before 7th administration		
	New method	Conventional method	Accuracy (%)	New method	Conventional method	Accuracy (%)	New method	Conventional method	Accuracy (%)
Eta-1	45.9	48.7	94.3	59.8	66.6	89.8	83.7	91.2	91.8
Eta-2	70.2	75.9	92.5	167.8	159.7	105.1	132.3	124.0	106.7
Eta-3	80.5	71.8	112.1	87.5	81.3	107.6	102.9	100.9	102.0
Eta-4	124.9	127.6	97.9	267.5	261.1	102.5	200.6	192.6	104.2
Eta-5	129.3	119.1	108.6	102.3	94.3	108.5	100.8	95.4	105.7
Eta-6	205.6	199.4	103.1	204.9	203.3	100.8	198.6	208.3	95.3

## Discussion

The freeze-drying method has been successfully applicated to the storage of biological samples of small chemical drugs. The biological samples of levetiracetam and mycophenolic acid were stored with the freeze-drying method after being obtained at the PK studies of both drugs in healthy volunteers^16^. It found that the detections of levetiracetam and mycophenolic acid were accurate and repeatable, and the analytes were maintained stability for a long time. The PK studies of levetiracetam and mycophenolic acid in healthy volunteers also showed that the PK parameters of analytes stored with the freeze-drying method were consistent with those stored with the conventional method. The degradation of drugs and plasma was prevented by inhibiting the activity of enzymes and retarding the growth of microbes as the biological samples of levetiracetam and mycophenolic acid were stored with the freeze-drying method. As for some proteins or polypeptides, the freeze-drying method may also help the proper conformations of analytes when samples were freeze-dried to remove the liquid during the freeze-drying process.

The detection of therapeutic proteins, including adalimumab and etanercept, was accurate and repeatable, and the analytes were stable for up to 14 days when samples were stored using the new method. Importantly, PK studies of adalimumab and etanercept in animals and humans were performed using this effective method. The results of these PK studies were consistent with those observed in other PK studies where samples were stored at low temperature after collection. All the above results indicate that the new method of sample storage allows accurate and repeatable detection of the analytes.

In order to prevent the degradation of analytes and to slow the deterioration of plasma, samples were conventionally stored at low temperatures, ranging from − 20 to – 80 °C, to inhibit the activity of enzymes and retard the growth of microbes^17,18^. Because liquid medium plays an essential role in the catalytic action of enzymes and the synthesis and utilization of nutrients of microbes, samples were conventionally frozen to restrict the role of liquid medium^19,20^. However, protein destabilization and aggregation may occur when the samples are frozen in storage or during the freeze-thawing step^10,11^. The storage of samples in the frozen state may subject the protein to a variety of destabilizing stresses including cryoconcentration of protein and co-solutes, protein denaturation at the ice-water interface, undesirable crystallization of co-solutes, pH shifts associated with buffer crystallization, and cold denaturation^21^. The above-mentioned phenomena may occur during storage in the frozen state, as well as during the thawing process, when interaction occurs between proteins and surfaces. Those phenomena also subject the protein to destabilization by causing the aggregation of misfolded or partially folded protein species^22^.

Therapeutic antibodies commonly undergo freeze-drying in the pharmaceutical industry^23,24^. The stability of the antibodies increases as degradation pathways and protein mobility are reduced in the dried state^25^. In the current study, the freeze-drying method was used for the sample storage of therapeutic proteins to prevent protein destabilization and aggregation that occurs during storage of liquid at temperatures below freezing or during thawing. After the samples underwent the freeze-drying process, ice was removed by sublimation in the primary drying stage, and water was removed by desorption in the secondary drying stage. Above mentioned problems that occur in the frozen state were mostly circumvented by the new storage method.

Biological samples of therapeutic proteins that are stored by the conventional method may require a cold chain during transport because therapeutic proteins in liquid may be prone to physical and chemical degradation^26^. Significant effort is required to ensure that the correct temperature (often reached to – 20 °C) is maintained during transport, and samples collected in clinical trials often require transport between different laboratories or research centers. A convenient storage method that has few special requirements for storage conditions may contribute to the efficient transport of samples. Compared to conventionally stored samples, freeze-dried samples can be easily transported without the need of cryogenic storage equipment because the analytes are stable for 14 days at 4℃. For this reason, the proposed storage method can facilitate the transport of samples when samples are prepared using the new method.

**Figure 4:**
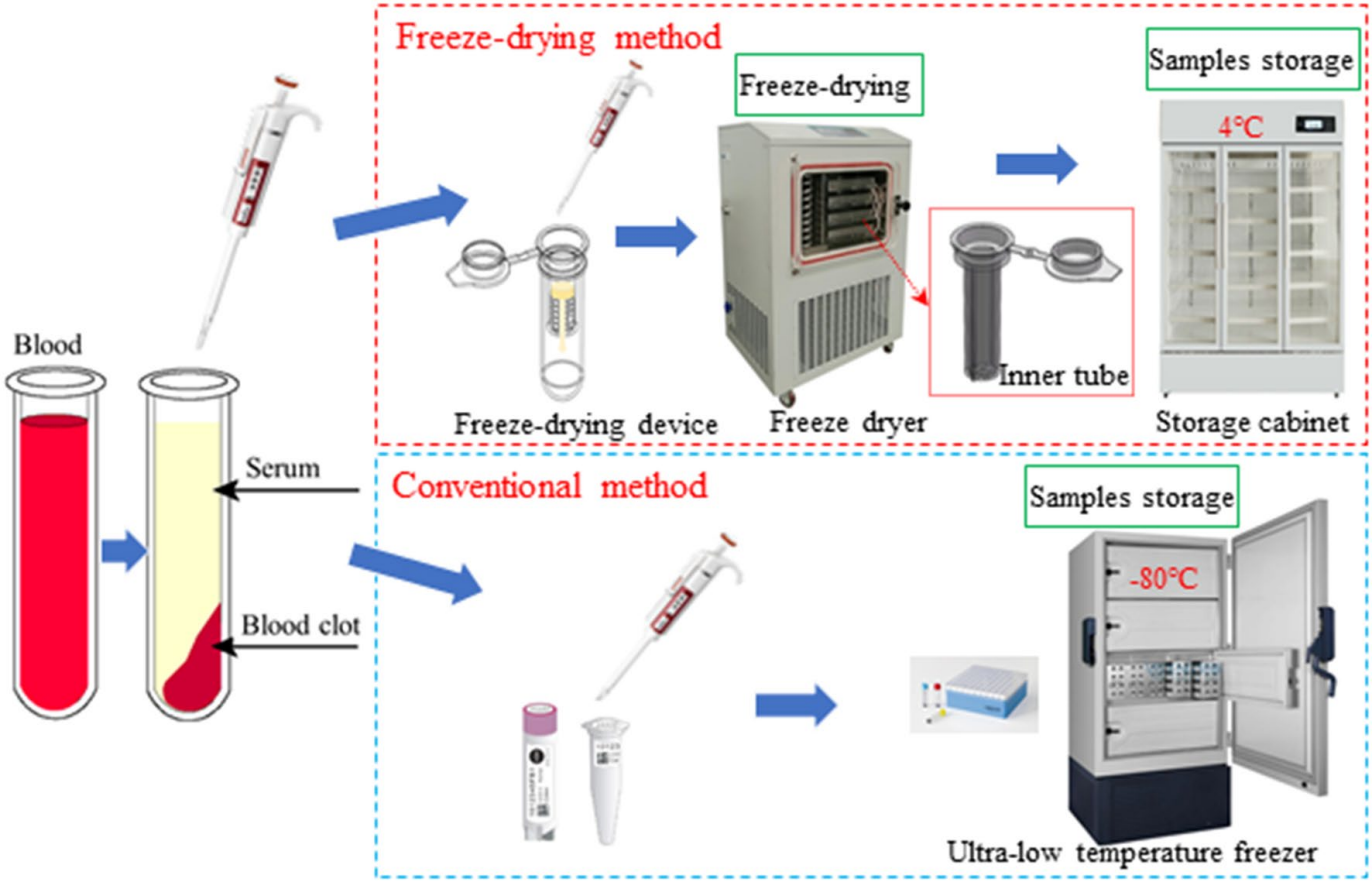
The serum samples were obtained after the blood was collected and maintained at 4℃ for 30 min. As for the freeze-drying method, serum samples were placed into the inner tube and ultrasonically blended for 5 min. The filtered sample flowed out from the inner tube and was reserved in the outer tube. The inner tube was then freeze-dried in a vacuum freeze dryer. After completing all programmed settings, the freeze-dried inner tubes were removed and eventually stored at 4℃ until analysis. Regarding the conventional method, the serum samples were transferred to polyethylene tubes and then stored in an ultra-low temperature freezer at – 80 °C until the time of the assay.

In addition to allowing convenient transport of samples, this new method is cost-efficient and easily applicable. In most study centers, early investment in the proposed method is approximately equal to that of the cost of an ultra-low temperature freezer. Using the proposed devices and method, medical staff and clinical researchers can easily freeze-dry samples, and therefore, this new method may become a practical option in clinical settings (Fig. 4). The practicability of this effective method has been validated by our PK studies of adalimumab and etanercept in animals and humans. Further testing of the method using other types of therapeutic proteins in human PK trials will be of significant value.

## Conclusion

The proposed method is effective for the storage and transport of biological samples of therapeutic proteins at 4℃ and does not require an environment that is below freezing temperature. Because this method enables ideal storage of therapeutic proteins while also conserving time, storage space, and energy, it will be attractive to most study centers for facilitating clinical and scientific drug research.

## Abbreviations

AS: Ankylosing spondylitis
ELISA: Enzyme-linked immunosorbent assay
PK: Pharmacokinetics
PBS: Phosphate-buffered saline
QC: Quality control
RSD: Relative standard deviation
RA: Rheumatoid arthritis
TMB: Tetramethyl benzidine
